# Core and cluster or head to toe?: a comparison of two types of curricula for teaching physical examination skills to preclinical medical students

**DOI:** 10.1186/s12909-024-05191-x

**Published:** 2024-03-26

**Authors:** LilyAnne Jewett, Samuel Clarke, Erin Griffin, Aaron Danielson

**Affiliations:** 1 Department of Medical Education and Clinical Sciences, Office of Accreditation, Assessment and Evaluation, Elson S. Floyd College of Medicine, Washington State University, Spokane, WA USA; 2 Department of Emergency Medicine, University of California at Davis, 2315 Stockton Blvd, PSSB 2100, 95817-2201 Sacramento, CA USA

**Keywords:** Physical examination, Medical students, Curriculum design

## Abstract

**Background:**

Despite the central importance of physical examination (PE) skills to patient evaluation, early trainees struggle with its correct application and interpretation. This struggle may reflect the instructional strategies of PE courses which have largely ignored the clinical reasoning necessary to accurately apply these skills. The “core + cluster” (C + C) is a recent approach to teaching PE to clerkship-level medical students that combines a basic ‘core’ exam with ‘cluster’ based on the student’s hypothesis about their patient’s clinical presentation. Our institution developed a novel C + C curriculum to teach PE to preclinical students. We aimed to assess the impact of this new curriculum on students’ clinical skills and course evaluations in comparison to the traditional “head-to-toe” approach we’d used previously.

**Methods:**

This was a retrospective study comparing two consecutive medical school cohorts exposed to the new (C + C) and prior (HTT) curricula respectively. We studied two complete cohorts of first-year medical students at our institution who matriculated in 2014 and 2015. The 2014 cohort received PE training via an HTT approach. The 2015 cohort received PE training via a C + C approach. Outcomes included performance scores on a statewide clinical performance exam (CPX) and student course evaluations.

**Results:**

We found no statistically significant difference in mean CPX scores between the two cohorts. However, student course ratings were significantly higher in the C + C cohort and students rated the C + C format as highly useful in clinical encounters.

**Conclusions:**

The C + C curriculum appears to be as effective a method of teaching PE to preclinical students as the HTT approach and is better received by students. We believe that this approach more appropriately reflects the way PE is used in clinical encounters and may help students with diagnostic hypothesis generation.

## Background

The physical examination (PE) is an essential component of most clinical encounters. Despite its centrality to helping clinicians form clinical hypotheses about patients’ clinical presentations, several studies have demonstrated a persistent lack of PE proficiency amongst undergraduate and graduate-level trainees [1–6]. This has resulted in renewed focus on how PE is being taught to preclinical medical students [7]. 

Historically, medical schools have taught PE using approaches that teach the PE as a comprehensive list of maneuvers to be performed by rote [8]. These approaches typically ignore the clinical reasoning that guides PE in clinical practice, layering it on after students master the psychomotor skills of PE. This has the potential disadvantage of teaching an overly detailed and undirected PE that is unwieldy in clinical practice. Clinicians do not perform comprehensive and undirected PE, but rather use it to gather data to support or refute clinical hypotheses based on their patients’ presentations. This lack of clinical context in teaching PE skills may actually hinder students’ effective application of PE in clinical settings, forcing them to “unlearn” what they have been taught.

Recently, Gowda et al. described a “core + clusters” (C + C) method as an alternate instructional design for teaching PE [9]. This approach was developed for clerkship-level students and describes a ‘core’ exam of 37 maneuvers that a clinician might typically perform on a patient requiring admission to the hospital. “Clusters” of related PE maneuvers can be added to the core exam depending on the clinician’s hypotheses about the patient’s clinical presentation (e.g., incorporating neurologic exam maneuvers for a patient with acute dizziness). This approach views clinical reasoning as a fundamental component of PE application.

In 2015, we developed and implemented a modified C + C curriculum for teaching PE to preclinical first-year medical students in our medical school, transitioning away from an HTT approach. We developed a novel “core” examination checklist for preclinical students which describes the PE maneuvers they might be expected to perform in a routine office visit. In similar fashion to the approach described by Gowda et al., we developed clusters of related PE maneuvers to augment our simplified core exam based on a patient’s chief complaint. Our goal was to develop a curriculum that would incorporate basic elements of hypothesis generation using PE and which could be understood by preclinical medical students. In June 2015, we piloted our curriculum with a small (*n* = 6) group of first-year medical students in an accelerated 3-year track within our medical school and found that the C + C curriculum was well-received and effective in preparing them for an end-of-course PE skills assessment as well as precepted patient encounters which are a part of our medical school’s curriculum. In August 2015 we implemented the curriculum for the entire first-year class, and it has been the introductory PE curriculum in use since that time.

To date, no studies have objectively assessed the effectiveness of the C + C approach in comparison to an HTT approach [7]. We aimed to objectively assess our new curriculum by comparing the performance of a cohort of first-year medical students exposed to the C + C curriculum to a cohort from the previous year who received the HTT curriculum. As our primary outcome, we chose overall performance scores on a statewide clinical performance examination (CPX). The CPX is a standardized test of PE and patient interviewing skills taken by medical students from 10 schools throughout California at the start of their fourth year of medical school. The development of the CPX and validity evidence supporting the exam have been previously described [10, 11]. We chose performance on the CPX sub-scores (history taking, physical examination, patient satisfaction, patient education, and patient interaction), and end-of-course evaluation scores as our secondary outcomes. We hypothesized that students exposed to our novel curriculum would demonstrate higher mean overall scores on the CPX and chose a priori a difference of greater than 10% as evidence of a potentially meaningful difference.

## Methods

### Study design

We conducted a retrospective cohort study comparing two classes of medical students at our institution; the class that matriculated in 2014 and which learned PE using the HTT method, and the class that matriculated in 2015 and which learned PE using the C + C method. This change in course format was the only major curriculum change that occurred during this time period at our institution, and we limited our analysis to these cohorts in order to avoid confounding from curriculum changes which may have occurred in the years prior to or subsequent to the intervention.

### Study setting

We conducted our investigation at UC Davis School of Medicine, a publicly funded medical school in Northern California. All participants were matriculated students participating in coursework at our main campus at the time of the research activity.

### Curriculum development/description of exposure

We drew upon multiple conceptual frameworks in designing our novel curriculum. Using the analogy of PE as a type of procedural learning, we incorporated Fitts and Posner’s theory of motor acquisition, Dreyfus’s model of skills acquisition, and Ericsson’s theory of deliberate practice to guide elements of the curriculum [12–14]. Learners were given an explicit framework of rules to follow and to guide decision-making around PE, and classroom time incorporated clinical cases and discussion as well as observed practice and directed feedback. Students were provided with three to four examples of complaints which each particular cluster exam would be indicated (e.g., cardiac and pulmonary exam for a patient with chest pain and cough). Gowda’s C + C approach provided a compelling and intuitive structure for approaching PE, but we recognized that it would need to be simplified and adapted to preclinical learners who would first be exposed to clinical medicine during an ambulatory care preceptorship.

### Derivation of novel core examination checklist

Gowda’s original core examination checklist was developed for clerkship-level students on an inpatient medicine rotation. We required a checklist that would fit the practice environment that our first-year students enter: the outpatient visit evaluating a well patient. This modified core examination would serve as a basic template for PE that could serve as the scaffold for our course.

We used a modified Delphi approach to address this challenge. Faculty from multiple specialties within our institution (Family Practice, Internal Medicine, General Surgery, Pediatrics, Emergency Medicine, Obstetrics and Gynecology, Psychiatry) were sent a prompt of a well-patient visit. Participants were asked to list the examination maneuvers that a first-year medical student should be expected to perform on such a patient. All maneuvers were binned by percentage of rater agreement into low (0–33%), moderate (34–66%) or high (67–100%) categories. All high-agreement items were included on the checklist.

We then conducted a focus group with core faculty from our longitudinal clinical skills course. They were instructed to add back any maneuvers with low- or moderate-agreement that they deemed essential for a well patient exam. Once consensus was reached, the checklist was finalized.

Our initial round generated 47 maneuvers, of which five generated high rater agreement: palpation for lymphadenopathy of the head and neck, cardiac auscultation, pulmonary auscultation, abdominal inspection, and abdominal palpation. Through the consensus of the focus groups, 10 items were added to the 5 items with highest agreement: general appearance, inspection of the eyes, inspection of the oropharynx, palpation of the lower extremities for edema, palpation of dorsalis pedis and posterior tibial pulses, forearm extension and flexion against resistance, hip flexion against resistance, and gait. A comparison of our novel core exam for preclinical students and the original core exam described by Gowda et al. is shown in Table 1.

**Table 1:** Comparison of the novel core examination for preclinical medical students to the original core examination for clerkship-level students described by Gowda et al.

Modified core examination for preclinical students	Original core examination described by Gowda et al.
General • In distress/no distress • “Sick/not sick” appearance Head • Eye inspection/pupillary light reflex • Oropharynx inspection Neck • Palpation for lymphadenopathy Thorax • Cardiac auscultation (4 areas with diaphragm and bell) • Pulmonary auscultation (6 areas posteriorly, 1 axillary, 1 apex bilaterally) Abdomen • Inspection • Palpation (superficial and deep) Extremities • Palpation for edema bilaterally • Dorsalis pedis and posterior tibial pulses bilaterally Neurological • Gross upper extremity flexion and extension against resistance • Hip flexion against resistance • Gait	General • General appearance • Level of consciousness • Orientation • Temperature • Weight/height/body mass index Vital Signs • Blood pressure • Heart rate • Respiratory rate HEENT • External inspection of eye and lid • Pupillary light reflex • Inspection of oropharynx and dentition Neck • Lymph node palpation • Thyroid palpation Chest • Thorax inspection • Chest auscultation (anterior and posterior) • Chest percussion posteriorly Cardiac • Carotid artery palpation • Jugular venous pulse • Cardiac point of maximal impulse • Cardiac auscultation with diaphragm in six areas • Cardiac auscultation with bell at apex Abdomen • Abdominal inspection • Abdominal auscultation • Abdominal palpation in six areas • Liver palpation • Lymph node palpation (inguinal) Vascular • Posterior tibial or dorsalis pedis artery palpation • Assessment of lower extremities for edema bilaterally Skin • Skin inspection Extremities and musculoskeletal • Inspection of extremities • Inspection of joints • Inspection of limbs for alignment and symmetry Neurological • Assessment of speech • Cranial nerves • Motor exam of upper and lower extremities (strength and tone) • Deep tendon reflexes (biceps and patellar) • Sensory exam (light touch or pinprick of feet) • Gait

### Cluster exams and course format

We developed cluster exams using a combination of organ systems and anatomic regions: pulmonary, cardiac, abdominal, neurologic, HEENT (head, eyes, ears, nose and throat), and male and female genitourinary exams. These cluster examination checklists were developed from prior organ system and body area checklists we used in our HTT curriculum. All checklists were reviewed by a multidisciplinary group of faculty with experience teaching physical examination to pre-clinical medical students. Due to their complexity, the head and neck and neurologic checklists were reviewed and revised with input from Otolaryngology and Neurology faculty, respectively. These checklists were not intended to be all encompassing, but rather to teach basic, essential maneuvers to novices. More advanced maneuvers could then be layered on during the second-year PE course.

**Fig. 1:**
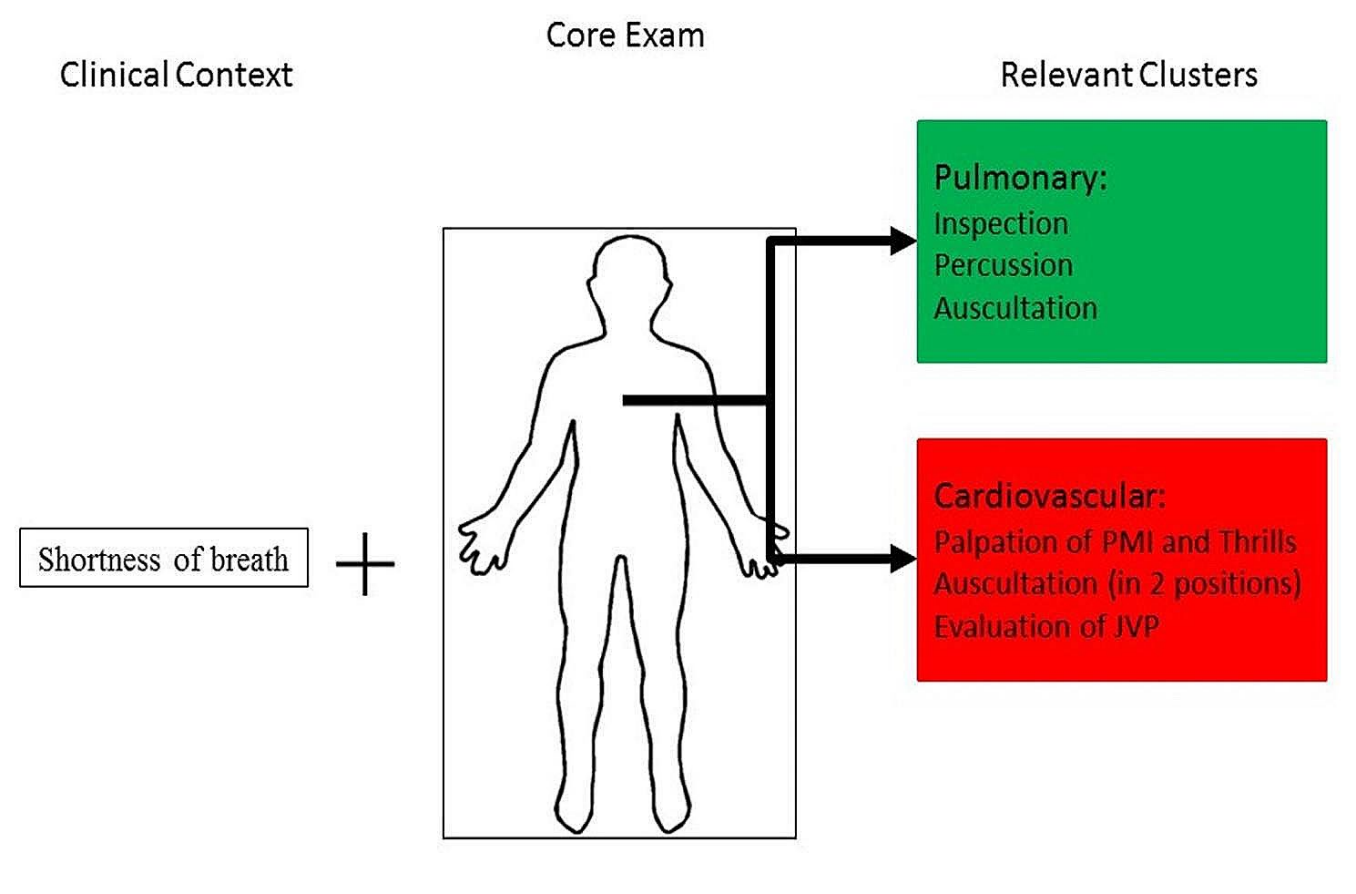
Novice decision making for core + cluster based on patient complaint

The course was taught over nine sessions, each 3 h in length. Each session framed the use of PE within a clinical case and included discussion of the rationale for specific PE maneuvers based on the patient’s complaint. Students were taught three symptom-based indications for each cluster exam which they were required to know for the course. Session time was then devoted to learning the micro skills relevant to the PE maneuvers covered in the session, and to peer-to-peer practice of PE skills with facilitator feedback. At each session students were given a period of time to practice the skills they had been taught. Each practice case started with a clinical vignette and required the students to choose the clusters indicated based on the complaint (Fig. 1). The students would then practice integrating these cluster examinations with the core exam. The C + C curriculum included an introductory session devoted to the core examination and was three hours longer in total than the previous HTT curriculum.

### Participants

This study included all students from the matriculating classes of 2014 (*n* = 99) and 2015 (*n* = 104).

### Bias

We included participants using an intention-to-treat principle, meaning that any students who extended their training or otherwise deviated from the standard curriculum track were included with the cohort they matriculated with. The pilot group of students from the accelerated 3-year track within our medical school were excluded from the study cohort.

### Data sources/measurement

We compared our two cohorts of students on the following pre-matriculation variables: undergraduate total and science grade point average (GPA) and Medical College Admission Test (MCAT) total score and sub-scores (biology, physical sciences and verbal). Post-intervention, we compared the summative end-of-course exam score and United States Medical Licensing Exam Step 1 to assess for differences between the two cohorts.

### Outcomes

We examined the following primary and secondary outcomes: total score on the California CPX (taken in the 4th year of medical school), CPX sub-scores (history taking, physical examination, patient satisfaction, patient education, and patient interaction), and end-of-course evaluation scores. The CPX consists of multiple standardized patient encounters and was developed by the California Consortium for the Assessment of Clinical Competence (CCACC), which our institution has been a member of since 2003. The exam is administered at each CCACC institution taken by every student at the start of their fourth year of medical school. At our institution, at the time these cohorts participated in the exam, students received a passing grade if their Overall Performance score is no less than 1.5 standard deviations (SD) below the mean, their subscores for History Taking, Physical Exam and Patient Education and Counseling were no less than 1.5 SD below the mean, and their subscore for Patient-Physician interaction was no less than 1.0 SD below the mean.

### Study size

Study size was limited to the classes that matriculated at UC Davis in 2014 (*n* = 99) and 2015 (*n* = 104).

### Statistical methods

We assessed differences using Chi-Square tests for categorical variables and independent two-tailed t-tests (*p* <.05) for continuous variables. We conducted a series of linear regression models to test: (a) whether performance in the course (regardless of curriculum) was associated with Step 1 or CPX performance (b) whether there was a cohort effect corresponding to the curriculum type the students were exposed to and (c) whether end-of-course evaluation scores were different between the two cohorts.

## Results

### Student performance

There were no statistical differences in baseline performance metrics between the two cohorts (Table 2). For the primary outcome of total performance on the CPX exam there was no statistical difference between the cohorts (Table 3). Differences in several secondary outcomes reached statistical significance. Course performance was slightly lower in the cohort exposed to the C + C curriculum (16.7 versus 17.1 for HTT cohort, *P* <.05; Table 2). CPX History scores were higher for the cohort exposed to the C + C curriculum (74.3 versus 72.3 for the HTT cohort, t = 2.5, *P* <.05). CPX Patient Satisfaction scores were higher for the cohort exposed to the HTT curriculum (90.6 versus 84.5 for the C + C cohort, t=-3.89 *P* <.05). USMLE Step 1 scores were not different between the cohorts. In unadjusted linear regression models, curriculum type was not a predictor of total CPX score. Curriculum type was a predictor of CPX History and Patient satisfaction sub-scores. However, the proportion of variance attributable to the curriculum type was minimal (R [2] of 0.03 for CPX History and 0.07 for CPX Patient satisfaction). There were no additional associations between curriculum type and CPX performance. There was no difference in CPX Physical Exam subscores between the cohorts and curriculum type was not a predictor of CPX Physical Exam subscore.

**Table 2:** Pre- and post-intervention metrics of students exposed to the head-to-toe versus core + clusters physical examination approaches

Phase	Metrics and Outcomes	Matriculation Year (n)	
		2014 (*n* = 99)	2015 (*n* = 104)
**I. Pre-metrics**	Undergraduate Total GPA	3.6	3.6
	Undergraduate Science GPA	3.5	3.5
	MCAT - Biology	10.8	10.9
	MCAT - Physical Sciences	10.3	10.1
	MCAT - Verbal	9.5	9.2
	MCAT - Total	30.7	30.1
**II. HTT and C + C**	Course Performance	17.1	16.7^1^
**III. Early Post**	Step 1 Score	219.7	221.3
**IV. Late Post**	CPX: History	72.3	74.3^1,2^
	CPX: Patient Satisfaction	90.6	84.5^1,3^
	CPX: Patient Education	67.3	69.8
	CPX: Patient Interaction	86.4	87.0
	CPX: Physical Exam	65.5	63.2
	CPX Total	75.5	76.1

^1^*t*-test, *p* = < 0.05

^2^ In unadjusted linear regression model, HTT and C + C was associated with a 2-point difference in CPX History scores, R^2^ = 0.03

^3^ In unadjusted linear regression model, cohort year was associated with 6-point difference in CPX Overall Satisfaction, R^2^ = 0.07

**Table 3:** End of course evaluation ratings of students exposed to the head-to-toe versus core + clusters physical examination approaches

End of Course Evaluation Questions and Scores (Likert Scale 1–7)	Cohort (Matriculation Year)						Delta	*t*
	2014			2015				
	*N*	Mean	SD	*N*	Mean	SD		
Overall rating of the course^1^.	72	5.38	1.1	78	6.08	0.9	0.70*	-4.14*
The course established and maintained a positive learning environment^2^.	72	5.82	1.2	78	6.46	0.8	0.64*	-3.78*
The course provided me with sufficient opportunities to self-assess the quality of my learning and identify areas that needed additional attention^2^.	72	5.56	1.3	78	6.05	1.1	0.49*	-2.54*
The course provided periodic useful feedback on my learning progress in specific areas^2^.	72	5.67	1.2	78	6.23	0.9	0.56*	-3.28*
The course stimulated and encouraged self-directed learning^2^.	71	5.51	1.4	78	6.18	1	0.67*	-3.42*

* Independent samples t-test, *p* <.05

^1^ 7-point Likert scale anchors: 1 = Poor; 7 = Excellent

^2^ 7-point Likert scale anchors: 1 = Strongly Disagree; 7 = Strongly Agree

### Course evaluations

See Tables 3 and 4 for course evaluation data. At the end of the novel C + C curriculum, students reported a high degree of confidence in performing the exam and rated the C + C method as useful in their clinical encounters (Table 4). The C + C curriculum was associated with higher ratings on all formal course evaluation questions (Table 3, *P* <.05 for all questions).

**Table 4:** Student ratings of comfort with the core + clusters approach and with its utility in clinical encounters

C + C Specific End-of-Course Evaluation Questions and Scores (Likert Scale 1–5)	C + C Cohort (Matriculation 2015)		
	*N*	SD	Mean
Rate how comfortable you feel performing each group of examination maneuvers: Core Examination^1^.	79	0.49	4.75
How satisfied were you with the physical examination portion of the curriculum^2^?	79	0.69	4.44
The core examination was a helpful tool when I went to the student run clinics or saw patients with my preceptor^3^.	79	0.8	4.56

^1^ 5-point Likert scale anchors: 1 = Not Comfortable; 5 = Totally Confident

^2^ 5-point Likert scale anchors: 1 = Very Unsatisfied; 5 = Very Satisfied

^3^ 5-point Likert scale anchors: 1 = Strongly Disagree; 5 = Strongly Agree

## Discussion

This is the first study to directly assess the efficacy of a novel core + clusters curriculum designed for preclinical medical students. We demonstrated that this C + C curriculum is as effective at teaching PE to preclinical students as the prior HTT method, though not superior in regard to performance on the CPX. Given the length of time between participants’ exposure to the curriculum and our selected outcome measures, this null finding may be due to washout of the training effect as well as the influence of confounders we were unable to measure. Nonetheless, the C + C curriculum was well-received by students, and we believe it warrants consideration as a teaching approach.

As described by Gowda et al., perhaps the greatest value of the C + C approach is the emphasis it places on diagnostic decision-making to guide the physical examination. Students are taught to consider a patient’s clinical presentation, and to apply appropriate cluster exams in order help them answer clinical questions (e.g., why is this patient short of breath?). This process stands in distinction to the traditional HTT approach, which treats PE as an undirected examination. As medical schools increasingly incorporate clinical experiences into the pre-clerkship years [15], an approach to teaching PE that accurately reflects the way it is used in clinical practice is essential.

While our novel PE curriculum was not shown to improve CPX performance, end-of-course evaluations showed higher student satisfaction compared to the HTT approach. In addition to increased overall satisfaction with the course, students expressed increased satisfaction with the new curriculum’s learning environment, opportunities for self-assessment, and opportunities for self-directed learning as compared to the HTT curriculum. Furthermore, the C + C curriculum cohort rated their comfort with performing the core exam (mean 4.75/5, *n* = 79), their satisfaction with the physical exam portion of the curriculum (mean 4.44/5, *n* = 79), and the core exam’s helpfulness in their early clinical experiences highly (mean rating 4.56/5, *n* = 79) (Table 4).

Given that this method emphasizes a focused exam in contrast to the HTT method’s comprehensive exam, some may claim that this approach allows for students to miss potentially significant physical exam findings [16]. While plausible, prior studies have suggested that graduating medical students’ poor physical exam performance lies primarily in application of physical exam skills, rather than in performance of a comprehensive exam [3]. These findings suggest that improvement in medical student physical exam skill may require a renewed focus on exam application rather than performance alone. Furthermore, it has been shown that experienced physicians rarely perform a comprehensive head-to-toe examination in clinical practice [5]. While the HTT exam is thorough, it is not a realistic or efficient use of time for most practicing physicians. The C + C approach aims to improve these deficits by teaching physical exam maneuvers in conjunction with consideration for PE application in various clinical scenarios. We believe that this approach better simulates a genuine clinical encounter and could potentially lead to a more focused and efficient patient interaction.

Our study is not without important limitations. We developed and conducted our intervention at our own institution, and with considerable support from our school of medicine. While there were no other major structural changes to the school of medicine curriculum between our control and intervention classes, there may have been changes in the training environment that we were unaware of and were unable to control for. Finally, we sought to measure the impact of this curriculum using an objective and well-validated measure of clinical ability: the CPX exam. While this outcome has the advantage of being psychometrically robust, the long interval of time between intervention and testing (over two years) makes washout of the teaching effect a likely possibility.

## Conclusion

In this single center cohort study, the C + C curriculum was equally effective to, and received superior learner satisfaction scores over the traditional HTT approach. The C + C approach is an important option for those looking for an alternative method for teaching PE skills.

## Data Availability

The data that support the findings of this study are available from the University of California at Davis School of Medicine but restrictions apply to the availability of these data, which are protected under the Family Educational Rights and Privacy act, and so are not publicly available. Data are however available from the authors upon reasonable request and with permission of the University of California at Davis School of Medicine. Please contact Erin Griffin at Erin.Griffin1@wsu.edu to request access to the data from this study.

## Abbreviations

PE: Physical Exam
C + C: Core and Cluster
HTT: Head to Toe
CPX: Clinical Performance Exam
HEENT: Head, Eyes, Ears, Nose and Throat
GPA: Grade Point Average
MCAT: Medical College Admission Test
CCACC: California Consortium for the Assessment of Clinical Competence
SD: Standard Deviation
